# Assessment of cerebral oxygenation response to hemodialysis using near-infrared spectroscopy (NIRS): Challenges and solutions

**DOI:** 10.1142/s1793545821500164

**Published:** 2021-06-17

**Authors:** Ardy Wong, Lucy Robinson, Seena Soroush, Aditi Suresh, Dia Yang, Kelechi Madu, Meera N. Harhay, Kambiz Pourrezaei

**Affiliations:** * Drexel University School of Bioengineering, Philadelphia, Pennsylvania; † Department of Epidemiology & Biostatistics, Drexel University Dornsife School of Public Health, Philadelphia, Pennsylvania; ‡ Drexel University College of Arts and Sciences, Philadelphia, Pennsylvania; § Department of Medicine, Drexel University College of Medicine, Philadelphia, Pennsylvania; ¶ Tower Health Transplant Institute, Tower Health System, West Reading, Pennsylvania

**Keywords:** Motion artifact removal, cerebral oxygenation, end-stage kidney disease, near-infrared spectroscopy

## Abstract

To date, the clinical use of functional near-infrared spectroscopy (NIRS) to detect cerebral ischemia has been largely limited to surgical settings, where motion artifacts are minimal. In this study, we present novel techniques to address the challenges of using NIRS to monitor ambulatory patients with kidney disease during approximately eight hours of hemodialysis (HD) treatment. People with end-stage kidney disease who require HD are at higher risk for cognitive impairment and dementia than age-matched controls. Recent studies have suggested that HD-related declines in cerebral blood flow might explain some of the adverse outcomes of HD treatment. However, there are currently no established paradigms for monitoring cerebral perfusion in real-time during HD treatment. In this study, we used NIRS to assess cerebral hemodynamic responses among 95 prevalent HD patients during two consecutive HD treatments. We observed substantial signal attenuation in our predominantly Black patient cohort that required probe modifications. We also observed consistent motion artifacts that we addressed by developing a novel NIRS methodology, called the HD cerebral oxygen demand algorithm (HD-CODA), to identify episodes when cerebral oxygen demand might be outpacing supply during HD treatment. We then examined the association between a summary measure of time spent in cerebral deoxygenation, derived using the HD-CODA, and hemodynamic and treatment-related variables. We found that this summary measure was associated with intradialytic mean arterial pressure, heart rate, and volume removal. Future studies should use the HD-CODA to implement studies of real-time NIRS monitoring for incident dialysis patients, over longer time frames, and in other dialysis modalities.

## 1. Introduction

Each year, over 100,000 people with end-stage kidney disease (ESKD) in the United States initiate renal replacement therapy, most commonly hemodialysis (HD), to stay alive. Although HD is a lifesaving treatment, it is also associated with adverse effects on the heart, gastrointestinal tract, limbs, and brain.^1^ With respect to neurologic function, imaging studies have linked HD with cerebral white matter damage,^2–7^ and numerous studies have shown that HD patients have elevated risks of stroke, depression, and cognitive impairment, particularly in frontal lobe functioning.^8–14^ Recent research has shown that HD induces declines in cerebral blood flow^15^ and that oxygen supply to the brain during HD is likely to be a key driver of neurologic outcomes among HD patients.^1,16,17^ However, traditional vital signs used to modify HD treatment (e.g., heart rate and blood pressure)^18–21^ are not direct measures of cerebral perfusion,^18,21–25^ which is regulated by numerous pathways that might be altered in the setting of ESKD.^26^ Therefore, there is a need for noninvasive tools that can directly assess the cerebral response to HD in real time, enabling clinicians to identify and intervene on HD-related cerebral hypoxia.

Near-infrared spectroscopy (NIRS) can be used to monitor variation in oxygen supply to the brain and has been identified as promising tool for use in HD settings.^27–29^ NIRS can penetrate tissue up to a few centimeters within the 700–900 nm optical window, corresponding to the pre-frontal cortex, when the probe is placed on the forehead. In this optical window, water is minimally absorbed to allow for maximum absorption of hemoglobin.^30^ This absorption attenuates the intensity of the light that scatters back to the surface. There is a relationship between changes in intensity of light measured on the surface and the concentration of oxyhemoglobin and deoxyhemoglobin in the tissue, permitting calculations to estimate changes in oxy and deoxy-hemoglobin concentrations using the modified Beer-Lambert law.^31^ Therefore, NIRS represents a noninvasive strategy to directly measure frontal lobe perfusion changes during HD. However, no research to date has examined how the unique features of HD patients or HD treatment might impact NIRS signals over long periods of monitoring. Therefore, more knowledge is needed before implementing widespread applications of NIRS for cerebral oximetry in clinical HD settings.

HD treatments are typically 4 h in duration and occur three times weekly. Treatments are characterized by regular patient movement and symptoms, including discomfort and cramping with fluid removal. Therefore, knowledge from existing NIRS studies of cerebral oxygenation, that have generally relied on commercial NIRS systems and/or assumptions of limited subject movement or short stimuli duration, might not be applicable in the setting of HD.^32^ Furthermore, the few studies that have specifically examined NIRS in HD have been in lighter-skinned patient populations (e.g., in Europe and Asia),^15,33–35^ whereas over 30% of in-center HD patients in the US are Black.^36^ Importantly, melanin attenuates near-infrared light and darker skin pigmentation can lead to biased oximetry measurements.^37,38^ Therefore, knowledge of NIRS performance in darker-skinned HD patients is a priority to support US-based clinical applications.

Acknowledging that HD is an important setting in which to establish options for cerebral oximetry monitoring, the central goal of this study was to identify and address the unique challenges for NIRS assessments during HD treatment in a predominantly African-American patient population. Our overarching objective was to design a novel NIRS signal processing algorithm, optimized for use in HD patients of all skin tones, that will enable artifact removal in real-time while detecting clinically meaningful signals of cerebral oxygen supply and demand mismatch during HD. Using the totality of the raw NIRS signal rather than the summary measurements generally available from commercial probes, we developed the HD cerebral oxygen demand algorithm (HD-CODA), a new NIRS data analysis methodology that is not reliant on typical assumptions of a steady NIRS baseline signal. We then tested the ability of the novel HD-CODA algorithm to distinguish between physiologic correlates of cerebral deoxygenation and nonphysiologic artifacts in a cohort of 95 HD patients.

## 2. Materials and Methods

### 2.1. Materials

We used multiwavelength LED (L6 * 730/850) from Marubeni for this study. We chose the OPT101 detector for its spectral responsivity in the near-infrared range (700–900 nm). We used a digital potentiometer (AD5171) and LED driver (AS1101) to adjust and drive the LED current, respectively.

### 2.2. Design of NIRS systems

We designed continuous wave 2 Hz NIRS systems in our laboratory using methods that have been described previously.^39^ There are three main components of the NIRS systems: (1) NIRS probes that consists of one multiwavelength (*λ* = 730 and 850 nm) LED and two OPT101 detectors. Each system consists of two probes, one on the left and the right side of the forehead; (2) a control box that operates the NIRS sensors; (3) a computer running the data collection GUI created in MATLAB for data acquisition and real-time plotting of the data. We calibrated the NIRS systems in this study using solid and liquid phantoms in accordance to pre-determined specifications.^40–42^

### 2.3. Study participant recruitment and testing

We recruited 100 participants from three Philadelphia-area dialysis facilities between July 2018 to May 2019. Participants were eligible for inclusion in the study if they were 21 years of age or older, English-speaking, receiving maintenance in-center HD three days per week, able to provide informed consent, and not visually impaired. After obtaining informed consent, participants were scheduled for NIRS testing during two consecutive HD sessions. In-center HD patients typically receive HD on one of two schedules: (1) Mondays, Wednesdays, and Fridays, or (2) Tuesdays, Thursdays, and Saturdays. Participants were scheduled for their visits on the first two days of their respective weekly treatment cycles (i.e., Monday and Wednesday, or Tuesday and Thursday). Participants arrived at the testing visits 30 min in advance of their treatment time. Upon arrival, they were fitted with specialized headbands containing the NIRS probes and underwent NIRS monitoring for 5–10 min to establish a pre-HD baseline. Study participants also completed a cognitive assessment on the first visit date with the CogState Brief Battery, a 15-min electronic cognitive assessment containing four card game-based cognitive tasks. For the purposes of this study, we focused on two of the four tasks that test frontal lobe function: Detection (a test of psychomotor function and processing speed), and Identification (a test of attention).^43,44^ This battery was chosen as the cognitive assessment instrument because of its reported test-retest reliability,^45–47^ ease of administration, and low requirement for verbal skills or educational attainment.^48,49^ The cognitive assessment was presented to participants on a laptop computer, under the supervision of a trained research coordinator. The research coordinator uploaded de-identified participant performance data to an online scoring system, which provides raw scores for each task as well as log transformed scores for statistical analysis and comparison with normative data. Participants were monitored with NIRS for the entirety of two consecutive HD sessions (approximately 8 h in total). Study personnel remained on-site throughout all HD sessions in a separate, nonclinical area to monitor the NIRS signal output and make adjustments as needed. Among 100 patients who provided informed consent to participate, five participants rescinded their consent to participate on the day of testing. Therefore, 95 study participants were included in the NIRS analyses. After three months, study participants repeated the computerized cognitive tasks to determine functional trajectories. The study protocol was approved by the Drexel University Institutional Review Board.

### 2.4. Design optimization for patient comfort

**Fig. 1.**
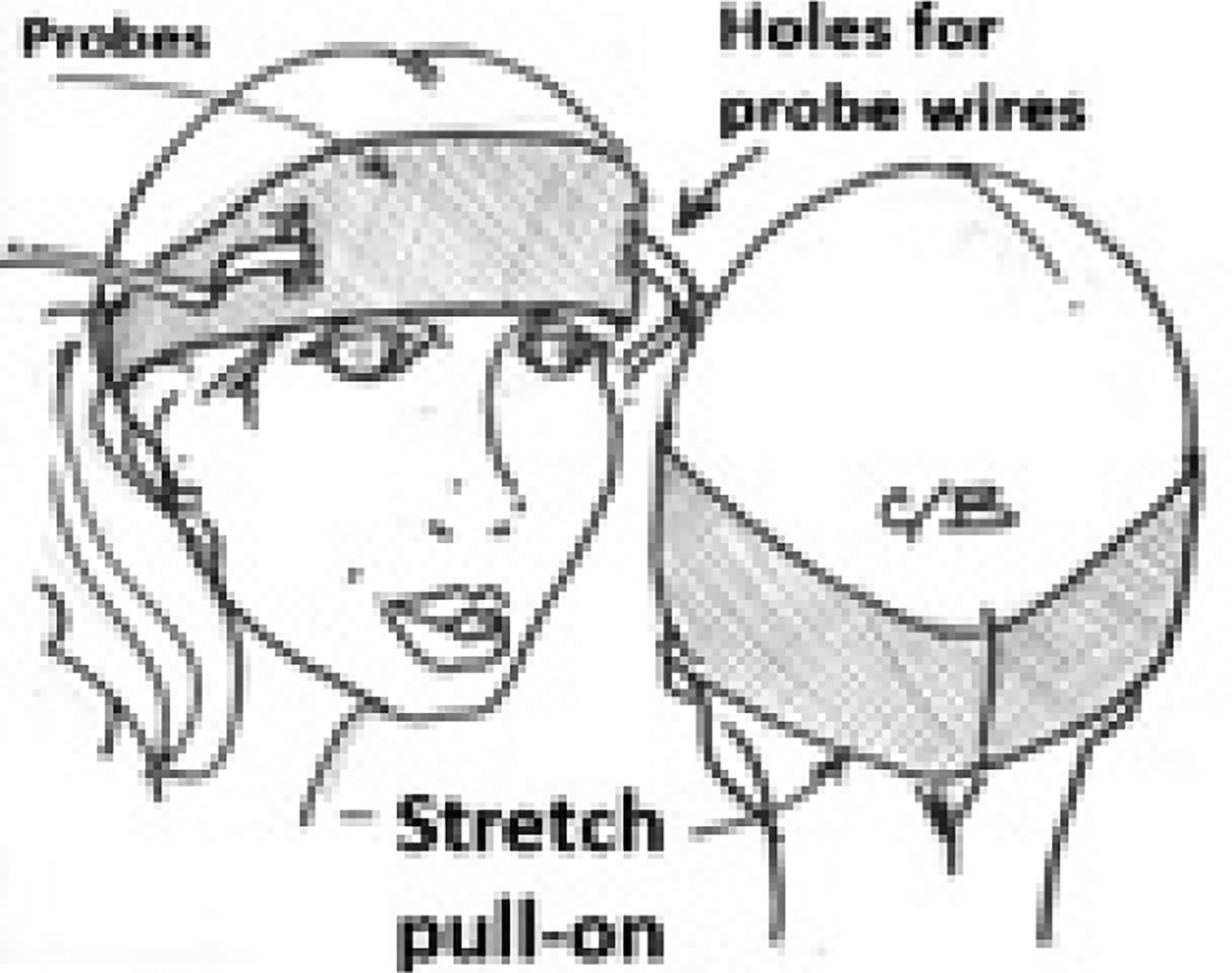
Diagram of customized headband that integrates a NIRS system for long-term wear.

Given that typical HD sessions are four hours in duration and HD patients commonly experience symptoms such as itchiness, sweating, and muscle cramping, we identified the need to optimize patient comfort while wearing the NIRS probe as a central priority. Based on patient feedback, we collaborated with a fashion designer from the Antoinette Westphal College of Media Arts and Design to create four different sizes of single-use headbands to ensure that the NIRS probe remained secured on patients’ foreheads while remaining comfortable (Fig. 1). Additionally, we used a flexible printed circuit board (PCB) consisting of a 0.1-mm thick polyimide flex material, which allowed the probes to fit the curvature of the patients’ foreheads.

### 2.5. Signal adjustments for darker skin

Melanin attenuates near-infrared light and research has shown that differences in skin pigmentation might lead to systematic bias in the assessment of oximetry in people with darker skin.^37^ Given that nearly one-in-three US individuals with ESKD is Black,^50^ we recognized that an important consideration when adapting NIRS to clinical HD settings was to ensure that the signals were reliable across the spectrum of skin pigmentation. We observed that the signal from our first probe testing a Black HD patient was very low in magnitude and too low to measure cerebral hemodynamic responses. Therefore, we replaced the LED of the probes from L4 * 730 * 850 to L6 * 730 * 850, as L6 has two more LED diodes per wavelength than the L4. However, in making this adjustment, we also observed increased variance and an amplified mean signal that necessitated specific signal processing considerations, described in Sec. 2.6.

### 2.6. Data processing approach

#### 2.6.1. Artifact removal

**Fig. 2.**
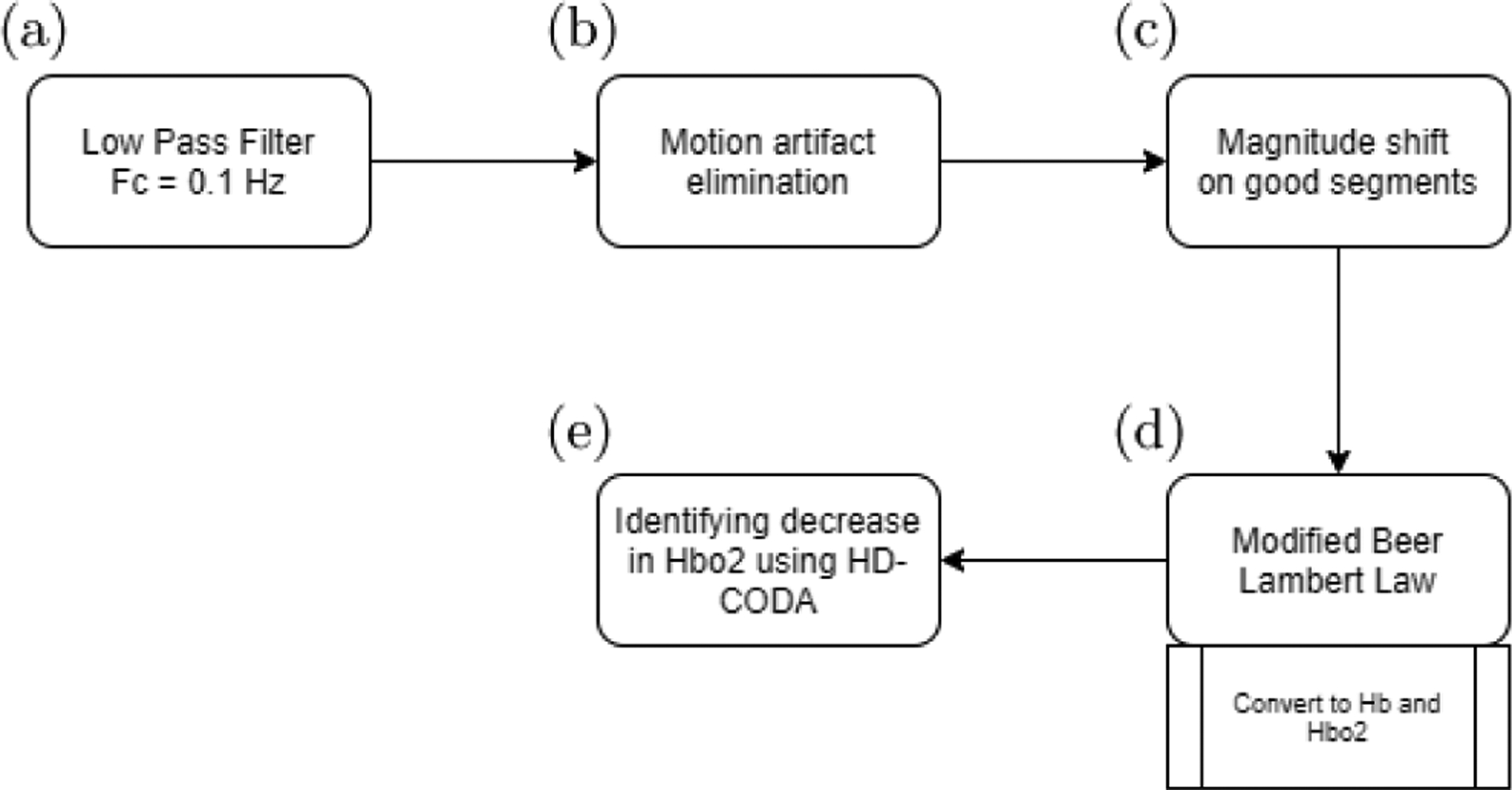
Flow diagram of NIRS signal pre-processing steps.

**Fig. 3.**
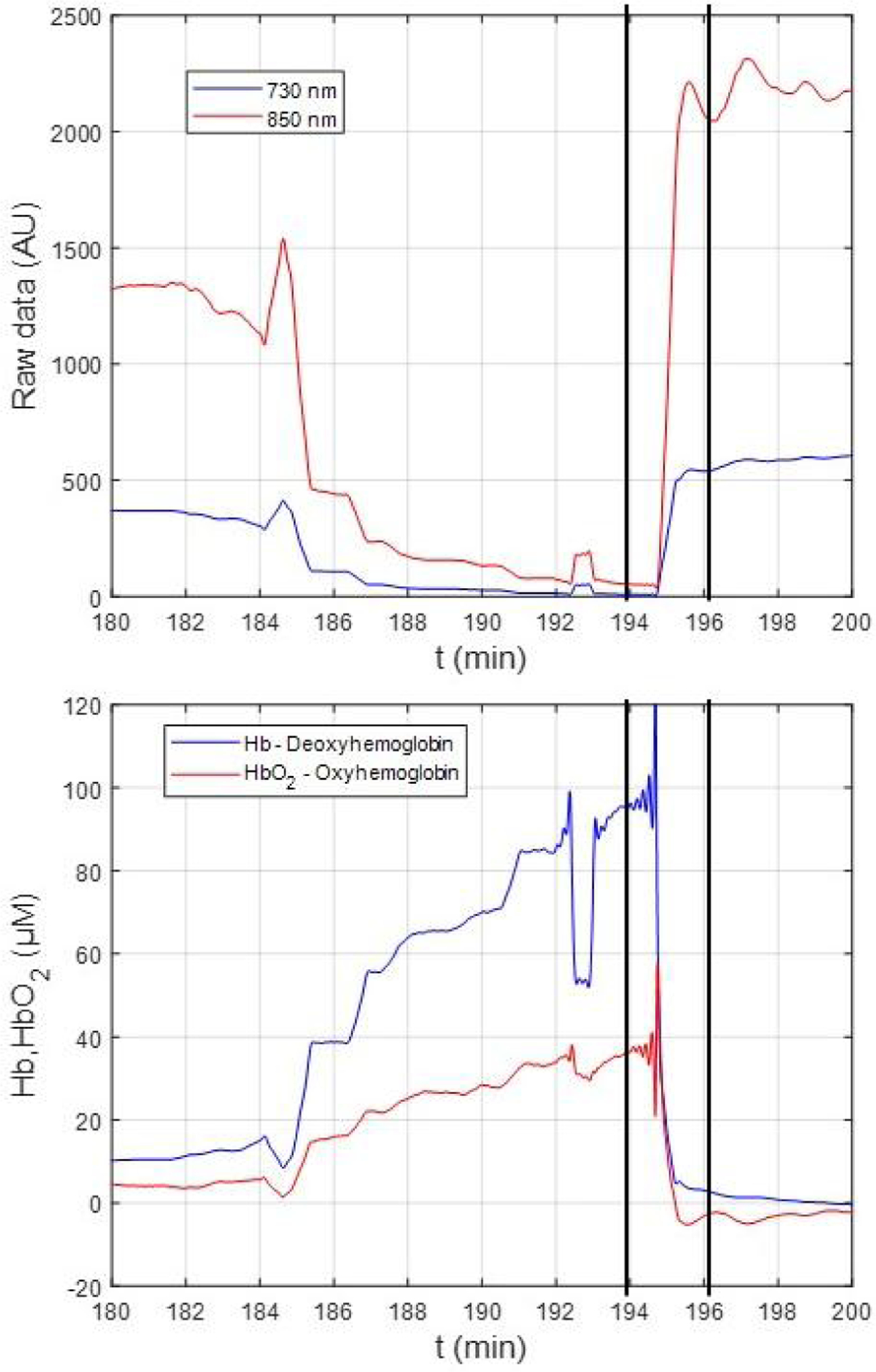
Example of motion artifacts indicated by the magnitude shifts in (top) both wavelengths, which resulted in a decrease in (bottom) both deoxy-hemoglobin (Hb) and oxy-hemoglobin (HbO_2_). Changes in the concentrations of HbO_2_ and Hb were calculated using the modified Beer-Lambert Law.^52^

**Fig. 4.**
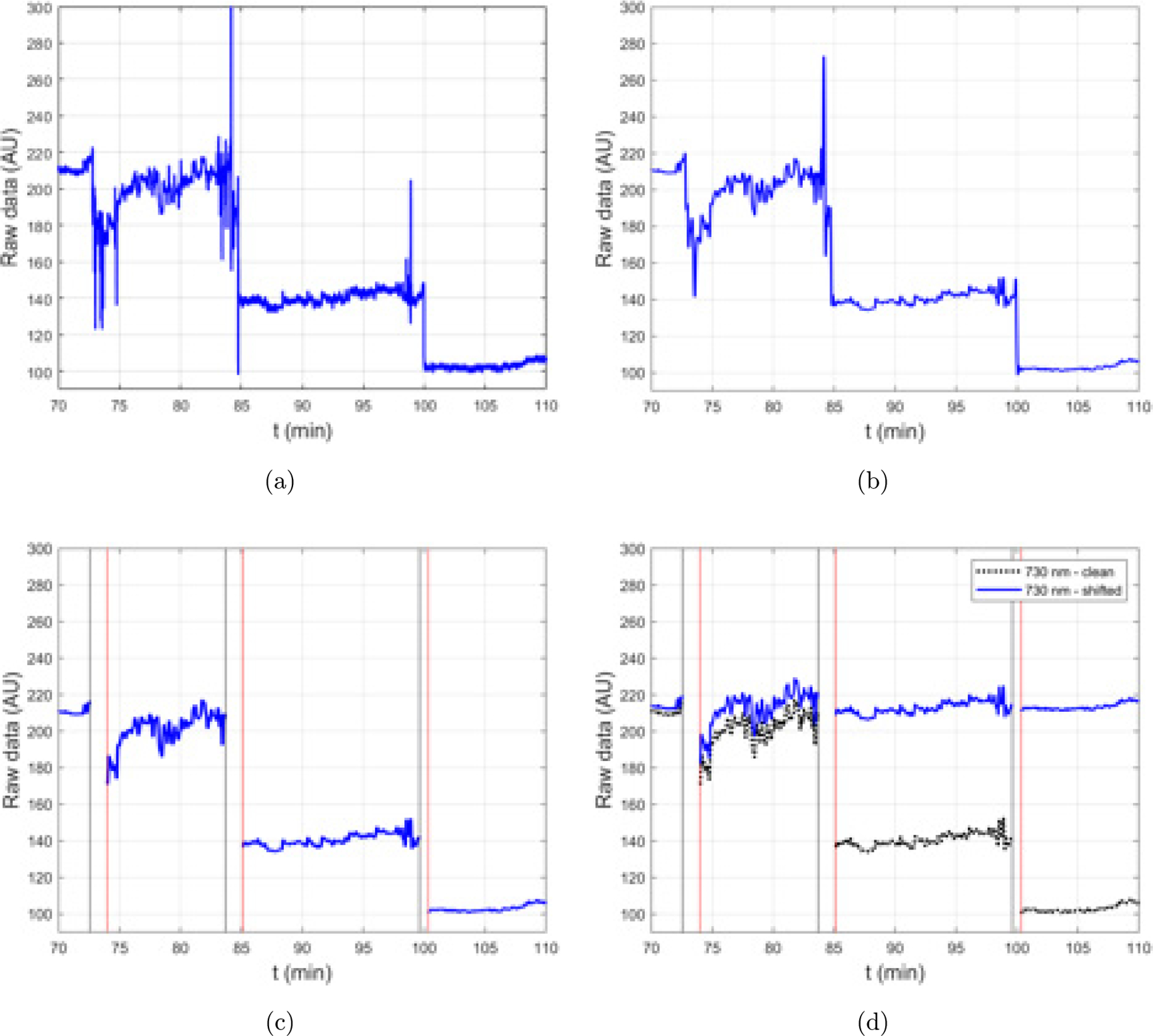
Example of data processing: (a) raw data, (b) low-pass-filtered data, (c) motion artifacts identified using the MARA algorithm, and (d) comparison between pre- and post-magnitude shift.

All data processing was conducted using MATLAB^™^ 2019b. Figure 2 displays a flow chart representing the stages of data processing and analysis. There were frequent magnitude shifts due to motion artifacts (Fig. 3). Each channel was visually inspected to reject any saturated or low signals. The accepted NIRS raw data was then filtered through a lowpass filter (fc = 0.1 Hz) to reduce high-frequency noise caused by cardiac or respiratory functions (Fig. 4). To remove motion and other artifacts and ensure quality and consistency of the data over long durations of wear, we adapted and modified the motion artifact reduction algorithm (MARA), created by Scholkmann and colleagues.^51^ The primary modification we made to Scholkmann’s method is that we did not transform and retain the portion of the signal identified as corrupt. Instead, we removed these data from the time series, replacing them with indicators for missing values. Upon visual inspection of the data, we believe that the data identified as motion and other artifacts did not contribute usable information and could not be made informative by applying smoothing transformations as in the standard MARA approach. In an additional modification, we used a moving average as well as a moving standard deviation (SD) to account for differences in amplitude between the 730 nm and 850 nm signals. To detect motion artifacts, we utilized the moving SD divided by the moving mean of the same window.

**Fig. 5.**
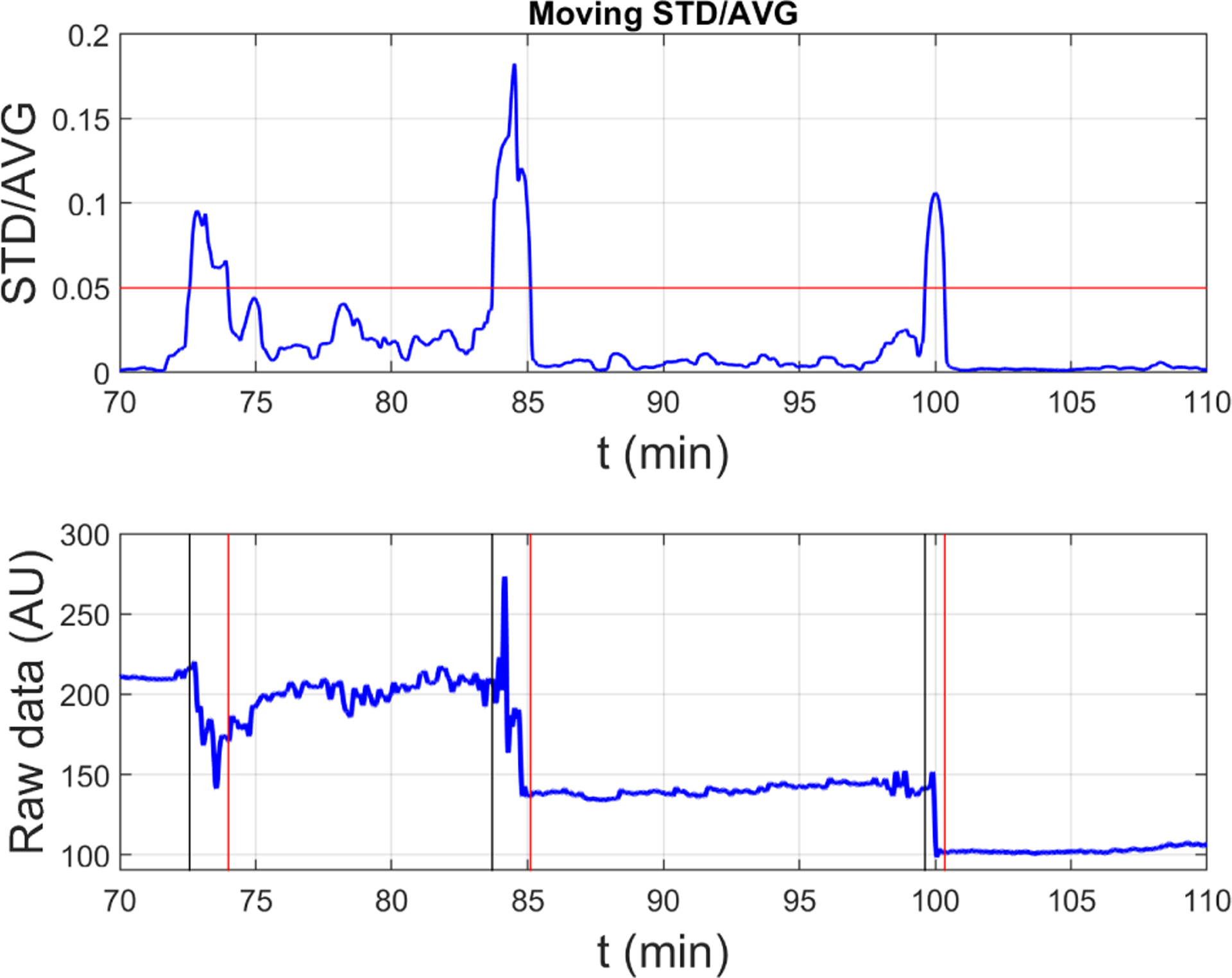
Figure showing the approach to address motion artifact and variation in between-subjects signal amplitudes using moving SD/mean (top) compared to raw data (bottom).

Our approach of using the moving SD/mean (Fig. 5) compensated for the differences in magnitude of NIRS raw data across participants. High motion or other compromised segments of the dataset were identified by applying a threshold to the ratio (moving SD over window [*t* − *w*, *t* + *w*])/(moving average over window [*t* − *w*, *t* + *w*]) for every time point *t* = *w* + 1, …, *t*_max_ − *w* and *w* = 120 s. After exploring a range of threshold values, and the possibility of subject-specific thresholds, a common value of 0.05 was selected to be used for all participants. We determine that this level was low enough to remove any egregious noise and high enough to not eliminate meaningful signals (Fig. 5).

We also observed a feature in the participant data that violated the assumptions implicit in standard measures of dynamic oxygenation which utilize an initial baseline time period: after motion events and other artifacts, we observed that the signal typically did not return to the previous baseline value. Specifically, we observed distinct shifts in the mean signal after periods of motion or other artifacts which were very unlikely to be due to changes in hemoglobin saturation (Fig. 3). Therefore, after artifacts are identified, our algorithm shifts the subsequent segment of good raw data (i.e., between identified motion artifacts) additively using the mean of the preceding 100 viable data points [Fig. 4(d)]. We used the first 60s of patient data, recorded before dialysis onset while patients were seated and still, to calculate Hb and HbOs using the modified Beer–Lambert law. Our technique for analysis, as described below, does not rely on comparison to an initial baseline, which is unlikely to be meaningful in long-duration cerebral oximetry of ambulatory HD patients.

#### 2.6.2. HD cerebral oxygen demand algorithm (HD-CODA)

Given the number of motion and other artifacts generated over long periods of NIRS probe wear during HD, it was a priority to develop automated methods that could identify oxygen supply/demand mismatch in real time while also minimizing artifacts. To address these challenges, we developed a novel algorithm, called the HD Cerebral Oxygen Demand Algorithm (HD-CODA). The challenge in identifying ischemic events was to distinguish them from nonphysiologic drift, which may remain in the data even after the most distinct artifacts are removed by the modified MARA algorithm described in the previous subsection. Additionally, given treatment times of 4 h on average and post-motion changes in the mean signal, it was important to define ischemic events without reference to an initial baseline period. To define time intervals that were consistent with the assumptions of real ischemic events but inconsistent with drift artifacts, we identified periods of time in which oxy-hemoglobin (HbO_2_) was decreasing and deoxy-hemoglobin (Hb) was increasing. The algorithm is described in detail below.

First, to identify intervals in which HbO_2_ is decreasing, we calculated a slope over time *β*_Hbo2_(*t*) in sliding windows of the discrete time series [HbO_2_(*t*)], *t* = 1, 2, …, *N* where:

- *N* is the total number of data points (each corresponding to 0.5 s for sampling frequency of 2 Hz),
- *β*_Hbo2_(*t*), *t* = 1, 2, …, *N* − *W* is the regression slope measuring the linear trend of the time series [HbO_2_(*t*), …, HbO_2_(*t* + *W* − 1)],
- *W* is the window length parameter.

**Fig. 6.**
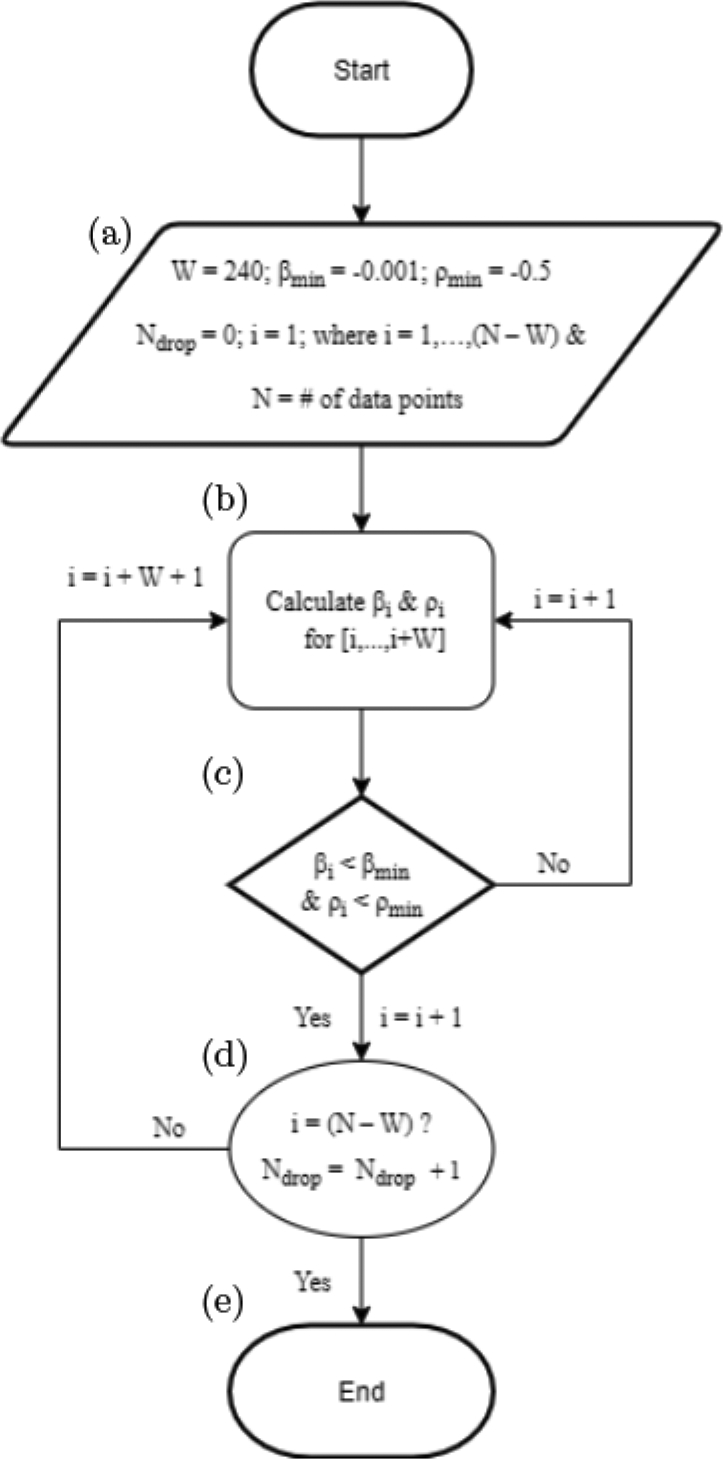
Flow diagram of ischemic condition algorithm (HD-CODA).

*β*_Hbo2_(*t*) is the average change in HbO_2_ (*μ*M) per second over the window [*t*, *t* + *W* − 1] [Fig. 6(b)]. Detected decreases in the HbO_2_ signal may be either physiologic or artifact-based. We distinguished between the two types of changes over time by identifying decreases in HbO_2_ that were accompanied by increases in Hb. Such changes were characterized by a negative correlation between HbO_2_ and Hb in addition to a negative slope of HbO_2_. Nonphysiologic signals typically exhibited HbO_2_ and Hb moving in the same direction over time, and were thus positively correlated.^53,54^ We calculated a moving correlation *ρ*(*s*)*, s* = 1, …, *N* − *W*, of the discrete time series HbO_2_(*t*) and Hb(*t*) over sliding windows of length *W*: *ρ*(*s*) = cor(Hb0_2_ (*s*, *s* + 1, …, *s* + *W*), Hb(*s*, *s* + 1, …, *s* + *W*)) [Fig. 6(b)].

Ischemic conditions were defined as time points where *β*_Hbo2_(*t*) < *β*_min_ and *ρ*(*t*) < *ρ*_min_, for set values of *β*_min_ and *ρ*_min_, which were parameters defined as described below [Fig. 6(c)]. To identify intervals of ischemia, we determined the start of each interval as the initial time point *t* for which the conditions *β*_Hbo2_(*t*) < *β*_min_ and *ρ*(*t*) < *ρ*_min_ were met for the time window [*t*, *t* + *W* − 1]. To determine the end of the interval, the beginning and end points of the sliding windows were shifted forward in increments of one-time unit until the ischemic condition criteria were not met. To account for differences in treatment times between participants and sessions, we defined *N*_drop_ as the number of data points identified within ischemic episodes during treatments (i.e., lasting two minutes or longer) as a proportion of total session time data points (*N*) to summarize oxygen supply demand mismatch across an entire session:

(1) $$\begin{array}{ll} {\%}_{\text{drop}} & =\frac{N_{\text{drop}}}{N};\\ N_{\text{drop}} & =\#\text{ of data points satisfying ischemic conditions definition within a session},\\ N & =\#\text{ data points in entire session} \end{array}$$

**Fig. 7.**
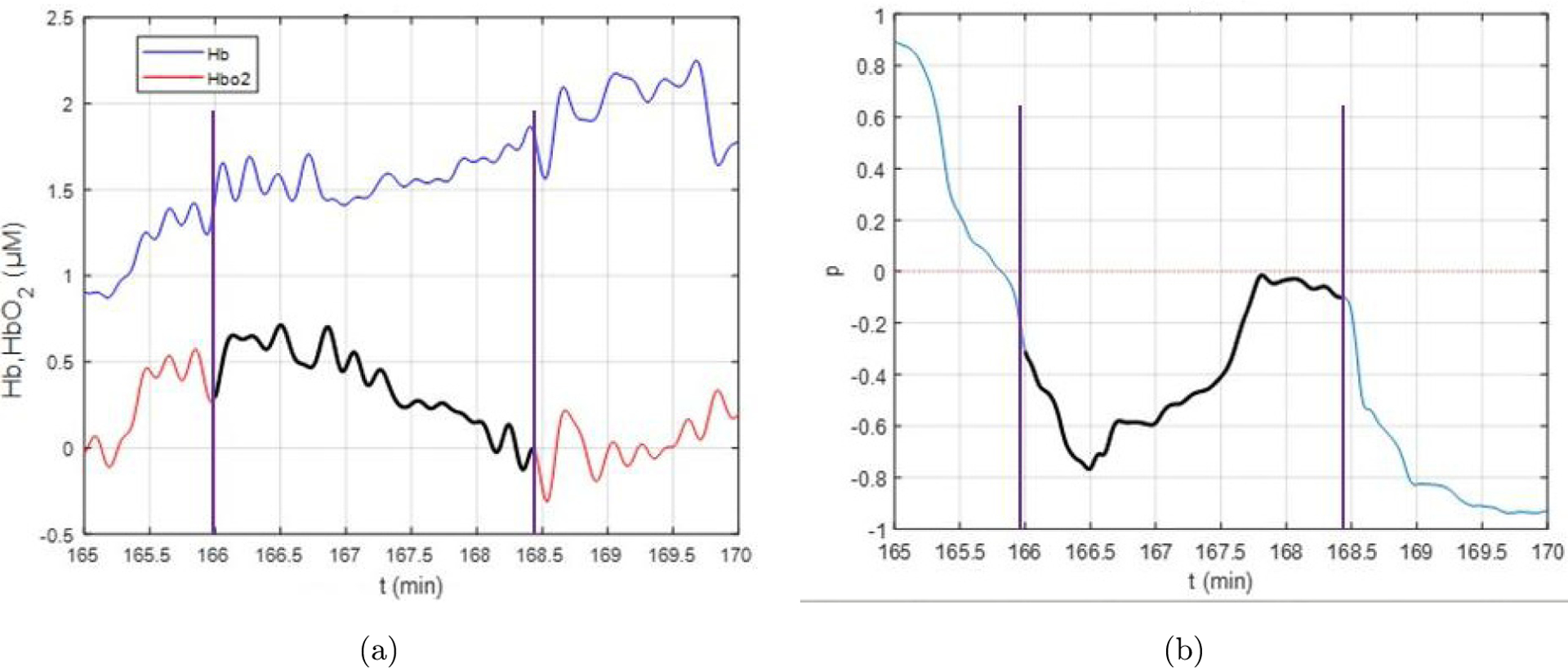
(a) Segment in black identifies when: *β*_Hbo2_(*t*) < *β*_min_ and *ρ*(*t*) < *ρ*_min_. (b) moving correlation between Hb and HbO_2_. Based on our chosen parameter values, a data segment with a *β* < *β*_min_ & *ρ* < *ρ*_min_ of at least 2 min duration was defined as an ischemic event (shown in black).

Using the parameters *W*, *β*_min_, and *ρ*_min_, the user can adjust the algorithm for a specific experiment. Our selected values are as follows:

- *W*: for too-small values of *W*, the algorithm may identify declines in HbO_2_ that are too fleeting to be clinically meaningful. For too-large values of *W*, the algorithm is more likely to fail to identify meaningful ischemic episodes. By visually evaluating the identified ischemic episodes for different values of *W* for several participants, *W* = 240 (2 min) was selected as the optimal value. This value was consistent with prior work describing two minutes of sustained cerebral desaturation as clinically meaningful in HD patients.^35^ Thus, the resulting algorithm identified any sustained ischemic episodes of at least two minutes duration (Fig. 7).
- *ρ*_min_: our goal was to detect ischemic conditions where HbO_2_ decreases and Hb increases, requiring evidence of a strong negative correlation. The value of −0.5 was chosen based on observed correlation over time intervals where ischemia events were identified by a trained analyst.
- *β*_min_: given our findings that a minimum of two min of sustained HbO_2_ decline was likely to identify clinically meaningful cerebral desaturation, we avoided values for *β*_min_ that were too small (more negative). Therefore, we tested values ranging from −0.001 *μ*M/s and −0.005 *μ*M/s. This range was chosen using a process of tuning the algorithm to detect episodes of decline in HbO_2_ in a sample of time intervals where ischemia events were identified using visual inspection of data by a trained analyst. A common value of −0.001 *μ*M/s was selected for all participants.

### 2.7. Associations between ischemic events derived by HD-CODA and HD treatment variables

We evaluated the association between a summary measure of observed cerebral ischemic time, calculated using HD-CODA, relative to total treatment time for each participant and physiologic variables expected *a priori* to have relationships with ischemia in the HD setting, namely those related to blood pressure and intensity of volume removal. Specifically, we examined associations between the proportion of time spent in a deoxygenation state during HD (i.e., %drop, Sec. 2.6.2) with summary measures of vital signs, such as average heart rate, average mean arterial pressure (MAP) and average and change in pulse pressure (PP) during the treatments. We also examined whether the summary measure could identify differences in cerebral perfusion between treatments with different inter-dialytic intervals (i.e., the first and second treatment of a weekly cycle), given the impact that longer interdialytic intervals can have on patient hemodynamics and volume status.^55^ Given that cerebral autoregulatory mechanisms maintain cerebral perfusion across a range of MAP, we expected a nonlinear association between MAP and the HD-CODA-derived measure, in which oxygenation changes are expected at the extremes of MAP (i.e., MAP < 60 and > 120 millimeters mercury).^35^ We expected similar nonlinear associations with PP, a marker of vascular response to intravascular volume shifts during HD.^56,57^

We calculated cerebral ischemia time relative to treatment time (i.e., %drop) for each participant using the fraction of total viable time points (after removal of corrupted data segments) that were identified as exhibiting ischemic conditions, based on the combination of a drop in HbO_2_ and an inverse relationship between HbO_2_ and Hb as described in the previous section (HD-CODA). MAP and PP are calculated from the diastolic blood pressure (DBP) and systolic blood pressure (SBP):

(2) $$\text{MAP}=\frac{\left(2*\text{DBP}+\text{SBP}\right)}{3}$$

(3) PP=SBP−DBP

Change in PP was calculated as:

(4) $$\Delta \text{PP}={\text{PP}}_{\text{post}-\text{HD}}-{\text{PP}}_{\text{pre}-\text{HD}}$$

We used a generalized linear model with binomial distribution to perform inferences on the overall ischemia outcome, measured as the fraction of total treatment time spent in the deoxygenated state. Specifically, we used a quasibinomial model which allows for differences in variance across subjects not explained by the binomial distribution parameters.^58^ For ease of interpretation, and because the relationships observed were approximately linear, we also computed linear correlations for comparison. Because we sought to establish associations between treatment ischemia time with hemodynamic variables that summarized the entire HD session, participant session data were included for these analyses only if 50% of the entire treatment session time had usable NIRS data. Therefore, sessions were excluded if, based on visual inspection by a trained analyst, a majority of the signal remained too low in amplitude, too saturated, or with too much motion artifact to be interpretable. The statistical analysis was done using the software R4.0.3.

## 3. Results

### 3.1 Description of study participant cohort

Among 100 participants who consented for the experiments, the median age was 58 years (interquartile range [IQR] 60–66), 92% were Black, and 49% were female. The median dialysis exposure time was 3.7 years (IQR 2.1–5.5 years) (Table 1). There were no significant differences in age, race/ethnicity, sex, or years on dialysis when comparing participants based on the number of sessions included in the summary ischemia analyses (Table 2).

### 3.2. Distribution of %drop as derived by HD-CODA

**Fig. 8.**
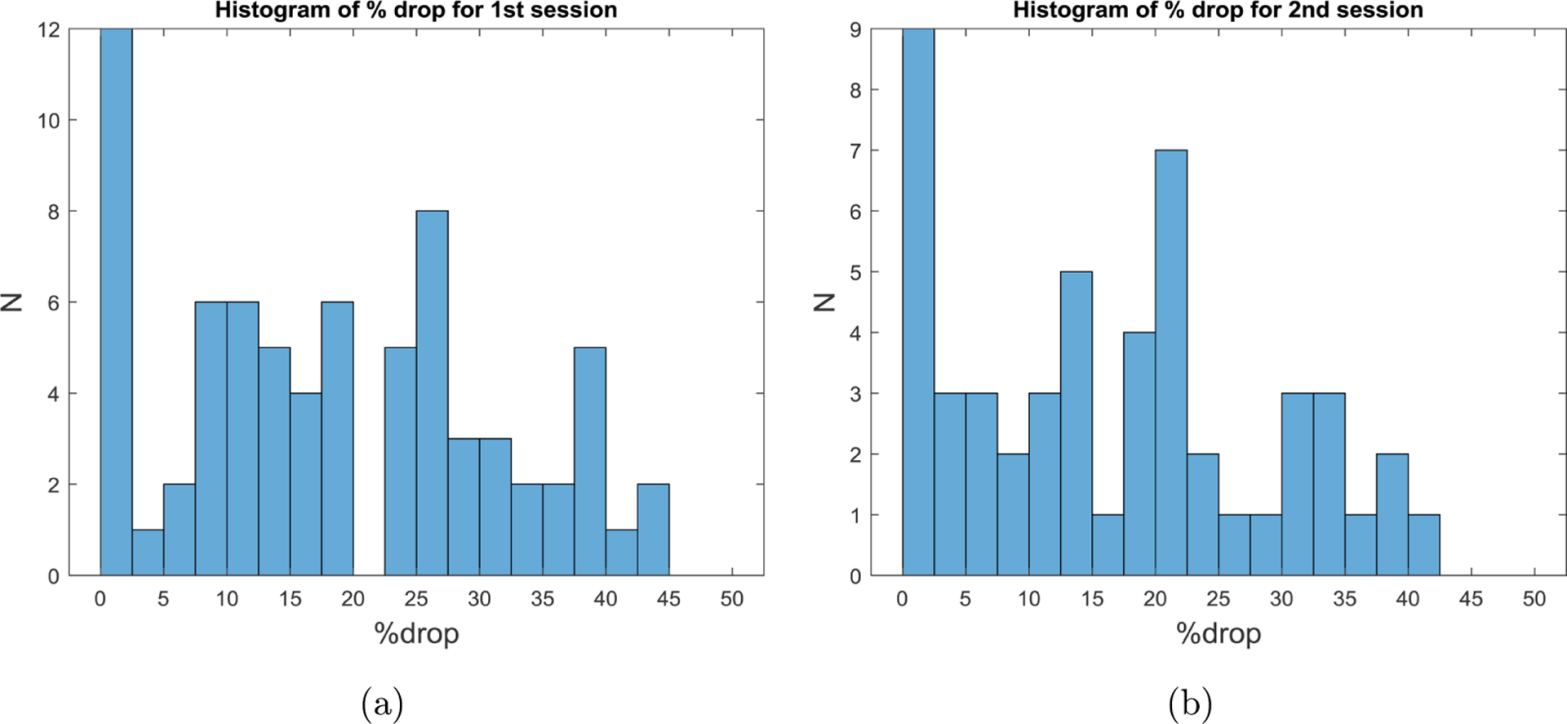
Histogram of %drop in (left) session 1, a treatment after a two-day interdialytic interval, and (right) session 2, a treatment after a one-day interdialytic interval. Values represent the proportion of total treatment time spent in cerebral ischemic conditions, as defined in Sec. 2.6.2.

Figure 8 displays the cohort distributions of %_drop_ (defined in Sec. 2.6.2) by treatment session. The proportion of treatment time spent in ischemic conditions (i.e., %drop) during HD session one (i.e., either Monday or Tuesday depending on patients’ schedules) was 19.2% ± 13.6%. Ischemic time relative to overall treatment time was slightly lower in the second HD treatment: 17.3% ± 13.2%.

### 3.3. Associations between %drop and summary clinical data

**Fig. 9.**
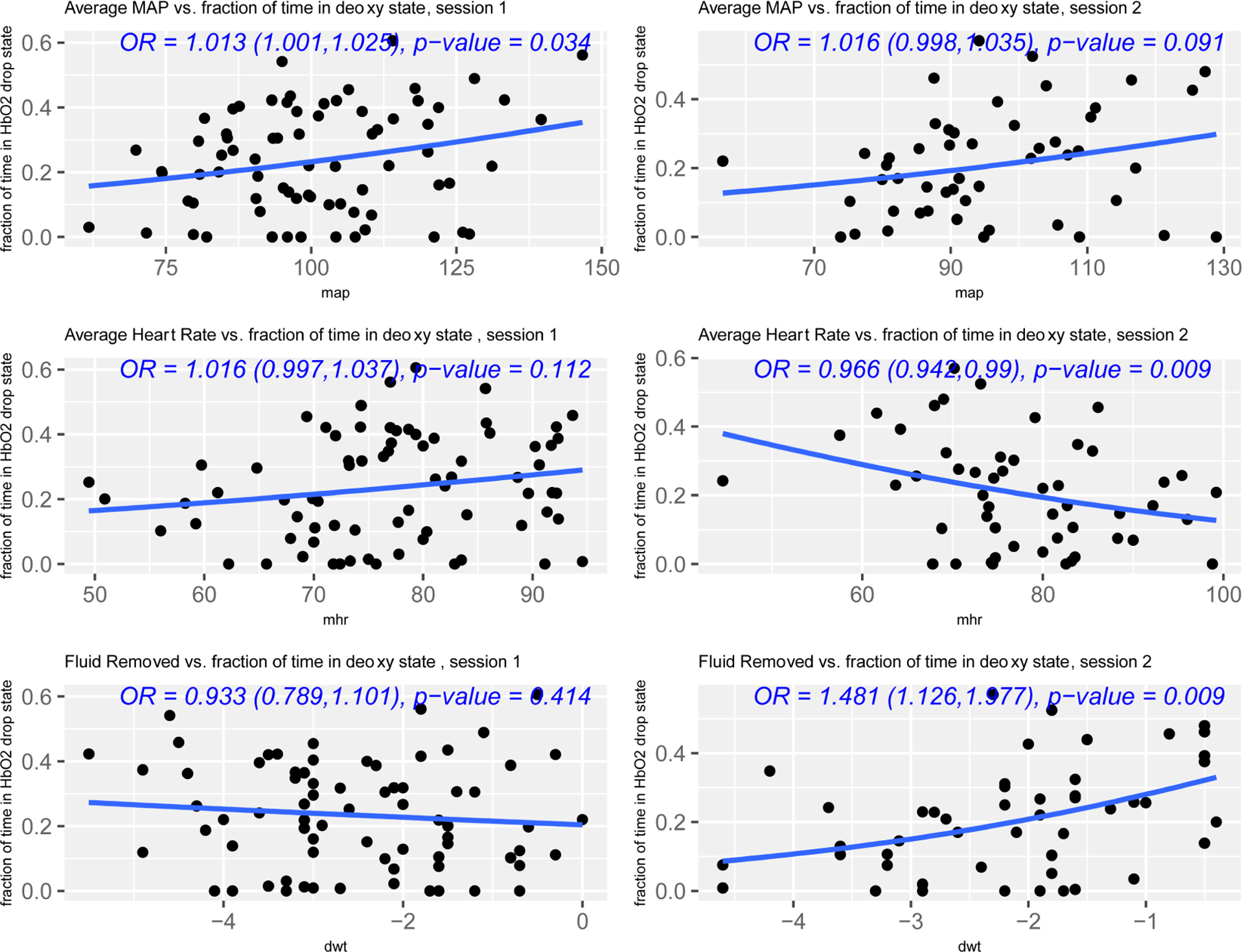
Associations between hemodynamic parameters and proportion of treatment time in deoxygenation conditions (i.e., % drop), (left) treatment session 1, and (right) treatment session 2.

We ascertained numerous hemodynamic and treatment-related measures throughout both HD sessions for all participants, including heart rate, blood pressure, blood flow rate, and ultrafiltration rate (in milliliters removed per hour, average 20 values per measure per participant over two treatment sessions). Tables 3 and 4 display summaries of the cohort distributions of hemodynamic measures in the first and second HD sessions, respectively. We observed a positive association between %drop and MAP for the 74 patients with viable data in session one. The relationship was approximately linear, and for descriptive purposes we computed a correlation of 0.25 between ischemia and MAP in the first session. For a test of association, using a quasibinomial generalized linear model we observed an odds ratio of 1.14 (95% confidence interval 1.01–1.28) corresponding to a 10-unit increase in MAP, with a *p*-value of 0.034. For session one, we did not observe significant associations between the summary ischemia measure and PP, patient age, and heart rate. For the second weekly session, there was strong evidence for associations between fraction of time in the deoxygenation state and heart rate, fluid removed during dialysis, and maximum ultrafiltration rate (Fig. 9). Table 5 displays correlation coefficients and corresponding *p*-values for each treatment measure and %drop. Cognitive performance on the Identification and Detection tasks was not significantly different between the initial study visit and the three-month follow-up visit.

## 4. Discussion

In this study, we used NIRS to assess the characteristic features of the cerebral hemodynamic response of patients undergoing HD and developed a novel algorithm to automatically detect episodes of cerebral oxygen supply demand mismatch during HD treatment. Using the novel HD-CODA algorithm, we calculated a summary measure of the proportion of time spent in deoxygenation conditions during HD treatment that was associated with hemodynamic and treatment variables and demonstrated differences in cerebral response to HD treatment in treatments with different interdialytic intervals. The results suggest that HD-CODA could be applied to process NIRS output in real-time during HD treatments, allowing clinicians to distinguish between artifacts and clinically meaningful signals of cerebral oxygen supply and demand mismatch that might require treatment modifications to prevent adverse neurologic outcomes.

Several recent studies have utilized imaging technologies ranging from magnetic resonance imaging (MRI),^59^ positron emission-computed tomography (PET-CT),^15^ and transcranial doppler^60^ to demonstrate that HD causes acute declines in cerebral blood flow and that these episodes of transient cerebral hypoperfusion are associated with higher risks of cognitive function decline. However, compared to these other imaging modalities, NIRS might be a more practical option to assess cerebral oximetry in clinical HD settings due to its ease of portability and lower associated costs.^29^ We optimized the comfort of wearing a NIRS probe during HD. Comfort is a priority given the long duration of HD treatments and burden of symptoms in HD patients. Leveraging our ability to examine the entirety of the raw NIRS signals from our cohort study, we identified numerous important challenges specific to the HD setting that should be addressed and overcome before implementation and interpretation of NIRS-based cerebral oximetry in HD patients.

One of the most important challenges we identified in the use of NIRS for HD cerebral oximetry monitoring was the introduction of artifact in the signal due to patient motion. In some cases, this artifact completely dominated our signal. We applied all known artifact algorithms and also developed HD-CODA to address these challenges. Furthermore, our study underscored the problems with relying on the assumption of a steady baseline signal to identify ischemia events. In a prior study of 58 prevalent HD patient in the United Kingdom, MacEwen and colleagues used a commercial NIRS system to assess the relationship between cerebral oxygenation during HD, blood pressure, and cognitive trajectory. Cerebral ischemia, defined in this study as a drop of 15% of baseline cerebral saturation for 2 min or more, was a common feature of dialysis treatments and associated with cognitive function decline.^35^ Similar to the study by McEwen and colleagues, HD-CODA also identifies ischemia events that are 2 or more minutes in duration. However, our ability to examine the entire raw NIRS signal also revealed that reliance on a baseline signal could be problematic in the analysis of real-time data in HD settings. We hypothesize that the observation of a gradual transition to a new steady state after motion artifacts was due to small displacements of the probe and gradual settling to a new position on the forehead. It should be noted that NIRS is extremely sensitive to probe positioning and motion and to the contact of source and detector with respect to the skin. Given that patient motion is unavoidable for long duration ambulatory treatments such as HD, HD-CODA and other strategies to eliminate baseline assumptions are needed.

The associations we observed between cerebral oxygenation and patient volume status, vascular tone, and dialysis intensity underscore the potential usefulness of real-time NIRS monitoring in HD settings. For example, to our knowledge, our study is the first to demonstrate how NIRS might provide important information on the hemodynamic tolerability of different treatments across the weekly dialysis cycle. First, we found that on average, ischemic episodes comprised a larger proportion of total treatment time in session one, which were treatments after a long (i.e., two-day) inter-dialytic interval), than in session two. These data are consistent with prior findings of an increased risk of stroke and other cardiovascular causes after long inter-dialytic intervals.^61^ Furthermore, we found that in session two, which is a mid-week session of HD, there was a strong negative association between volume removal and proportion of time in deoxygenation conditions — a relationship that was not observed in session one. This finding might indicate that volume removal is more likely to precipitate cerebral oxygen supply demand mismatches when HD patients are euvolemic than when they are hypervolemic (i.e., after a long interdialytic interval). Alternatively, the findings could signal that the tolerability of volume removal in the mid-week session is related to cerebral oxygen supply demand mismatch. These findings have important implications for clinical practice, as a clinically relevant, direct measure of cerebral oxygen demand during HD would enable timely treatment modifications to prevent neurotoxicity.

This study has several strengths, including its cohort characteristics and use of the raw NIRS signals for all study participants. This study also has several limitations that should be considered when assessing the findings and designing future research. We did not observe substantial hypotension during the treatment sessions and there was a lack of significant change in cognitive function after the three-month follow-up period, limiting our ability to detect associations between more extreme hemodynamic shifts, cerebral perfusion, and cognitive trajectories. Studies are needed of older HD patients and incident HD patients (i.e., those who are newly initiating HD) because patients might be more likely to experience dramatic volume shifts and cognitive declines in their first year of HD than in subsequent years of treatment.^62,63^ Furthermore, given an increasing national emphasis on transitioning in-center HD patients to home therapies,^64^ studies are needed to understand the utility of NIRS to monitor cerebral oximetry of patients who rely on peritoneal dialysis or home HD, which are modalities that are typically longer in duration but better hemodynamically tolerated. Studies using accelerometers on the NIRS probes are also warranted to further examine the influence of motion artifact during HD on NIRS signals. Future studies might also include more treatment measurements and flexible data analysis methods using information from the data beyond the fraction of time in the deoxygenated state, perhaps using machine learning methods to determine which HD-CODA features are most strongly associated with adverse neurologic outcomes in HD.

## 5. Conclusion

In this large, diverse cohort study of HD patients in which we assessed cerebral oximetry using NIRS over two treatment sessions, we identified and addressed several challenges specific to using this technology in the HD treatment setting. The primary challenges that we addressed in this study were related to patient comfort wearing the probe over long durations, the signal attenuation observed when assessing oximetry in people with darker skin, and the substantial motion artifacts that occurred regularly throughout treatment sessions which violated assumptions of a stable baseline signal. To respond to these challenges, we adapted existing techniques for signal processing and developed a novel technique called HD-CODA that automates signal processing to detect ischemic events. In future studies, we hope to apply our methodology to studies of cerebral oximetry in incident HD patients who are at the highest risks of substantial hemodynamic shifts and cognitive decline.

## Figures and Tables

**Table 1.** Cohort Characteristics, *n* = 100.

Characteristic	
Age, median (IQR)	58.3 (49.6, 66.0)
Sex	
Male	51 (51%)
Female	49 (49%)
Race	
NonHispanic White	2 (2%)
NonHispanic Black	91 (92%)
Hispanic	6 (6%)
Other	1 (1%)
Access Type	
Catheter	11 (11%)
Fistula	50 (50%)
Graft	39 (39%)
Years on Dialysis	3.65 (2.09,5.48)
Educational Attainment	
Less than High School	28 (28%)
High School Graduate	37 (37%)
Some College/Technical School	19 (19%)
College Graduate	16 (16%)
Cause of ESKD	
Type I Diabetes	1 (1%)
Type II Diabetes	39 (39%)
Hypertension	42 (42%)
Other	18 (18%)
Hemoglobin g/DL, median (IQR)	11.3 (10.5, 11.8)
Albumin g/DL, median (IQR)	4.0 (3.8, 4.2)

**Table 2.** Comparison of cohort characteristics stratified by the number of sessions included from participants in the summary ischemia measure analyses. Sessions were included if there was a usable NIRS signal for ≥ 50% of total treatment time.

Characteristic	No missing sessions *N* = 50	One missing session *N* = 26	Two missing sessions *N* = 19	*p*-value
Age, median (IQR)	58.3 (49.7, 65.3)	57.1(49.8, 67.5)	59.9 (54.1, 66.3)	0.98
Sex				
Male	25 (50%)	14 (54%)	10 (52%)	0.85
Female	25 (50%)	12 (46%)	9 (47%)	
Race				
NonHispanic White	0 (0%)	1 (4%)	1 (5%)	0.54
NonHispanic Black	48 (96%)	23 (88%)	16 (87%)	
Hispanic	2 (4%)	2 (8%)	1 (5%)	
Other	0 (0%)	0 (0%)	1 (5%)	
Years on Dialysis	4.0 (2.1, 6.2)	3.4 (1.9, 4.8)	3.0 (2.5, 4.3)	0.14

**Table 3.** Distribution of hemodynamic variables, Session 1.

Average MAP (mmHg)	Average pulse pressure (mmHg)	Change in pulse pressure (mmHg)	Average heart rate (BPM)	Maximum ultrafiltration rate (mL/hour)	Weight change (kg)	Difference in log-transformed cognitive scores over 3 months, identification task	Difference in log-transformed cognitive scores over 3 months, detection task
Min.: 62	Min.: 27	Min.: −77	Min.: 50	Min.: 85	Min.: 0	Min: −0.19	Min: −0.33
1st Qu.: 87	1st Qu.: 49	1st Qu.: −24	1st Qu.:70	1st Qu.: 580	1st Qu.: −1.5	1^st^ Qu: −0.06	1^st^ Qu: −0.09
Median: 98	Median: 60	Median −9	Median:77	Median: 757	Median: −2.4	Median: −0.02	Median: −0.02
Mean: 100	Mean: 62	Mean −8	Mean: 77	Mean: 815	Mean: −2.4	Mean: −0.009	Mean: −0.0039
3rd Qu.: 111	3rd Qu.: 74	3rd Qu.: +8	3rd Qu.: 84	3rd Qu.: 1000	3rd Qu.: −3.2	3^rd^ Qu: 0.03	3^rd^ Qu: 0.04
Max.: 147	Max.: 117	Max: +57	Max.: 98	Max.: 1880	Max.: −5.5	Max: 0.37	Max: 2.86

*Notes*: Abbreviations: mmHg — millimeters mercury; BPM — beats per minute; mL — milliliters; kg — kilogram

**Table 4.** Distribution of hemodynamic variables, Session 2.

Average MAP (mmHg)	Average pulse pressure (mmHg)	Change in pulse pressure (mmHg)	Average heart rate (BPM)	Maximum ultrafiltration rate (mL/hour)	Weight change (kg)
Min.: 57	Min.:28	Min.: −87	Min.: 45	Min.: 0.53	Min.: 1.1
1st Qu.: 88	1st Qu.:46	1st Qu.: −17	1st Qu.:70	1st Qu.: 500	1st Qu.: −1.4
Median: 95	Median: 59	Median: −8	Median: 76	Median: 700	Median: −2.0
Mean: 97	Mean: 58	Mean: −8	Mean: 77	Mean: 691	Mean: −2.1
3rd Qu.: 107	3rd Qu.: 68	3rd Qu.: +2	3rd Qu.: 84	3rd Qu.: 877	3rd Qu.: −2.9
Max: 131	Max: 97	Max.: +37	Max: 99	Max.: 1310	Max: −5.0

*Notes*: Abbreviations: mmHg — millimeters mercury; BPM — beats per minute; mL — milliliters; kg— kilogram

**Table 5.** Correlations between hemodynamic parameters and %drop for session 1 and session 2.

	Correlation with %drop (*p*-value)	
Variable	Session 1	Session 2
Average MAP (mmHg)	0.25 (0.033)	0.24 (0.09)
Change in pulse pressure (mmHg)	0.05 (0.67)	0.02 (0.91)
Average pulse pressure (mmHg)	−0.06 (0.58)	0.09 (0.53)
Average heart rate (BPM)	0.19 (0.11)	−0.36 (0.009)
Maximum ultrafiltration rate (mL/hour)	0.14 (0.25)	−0.45 (0.0009)
Weight change (kg)	−0.10 (0.41)	0.38 (0.007)
